# Treatment evaluation of Wharton’s jelly-derived mesenchymal stem cells using a chronic salpingitis model: an animal experiment

**DOI:** 10.1186/s13287-017-0685-0

**Published:** 2017-10-17

**Authors:** Zhe Li, Zhao Zhang, Xin Chen, Juan Zhou, Xiao-min Xiao

**Affiliations:** 1 The Department of Obstetrics and Gynecology at the 1st Affiliated Hospital of Jinan University, Guangzhou, 510000 China; 2 The Department of Reproduction at the Southern Medical University Affiliate Dongguan People’s Hospital, Dongguan, China

**Keywords:** Chronic salpingitis, Wharton’s jelly-derived mesenchymal stem cells, New Zealand rabbits

## Abstract

**Background:**

The present study was conducted to evaluate new methods to repair the reproductive function of the oviduct, thereby allowing gametes to combine and grow in vivo under natural circumstances.

**Methods:**

Sixty pathogen-free female New Zealand rabbits were divided into three groups: a wild-type group, an untreated control group, and a treatment group. Disposable sterile newborn sputum suction tubes were inserted into the urogenital tract to instill an *Escherichia coli* suspension into the uterine cavity to establish the chronic salpingitis model. Wharton’s jelly-derived mesenchymal stem cells (WJMSCs) or normal saline were used to treat this infection via different methods. The therapeutic effect was assessed by evaluating morphology, inflammatory factors, proteinology, and pregnancy outcomes.

**Results:**

Oviducts of New Zealand rabbits in the untreated control group showed structural failure and abnormal supermicrostructure of epithelial cells. WJMSCs could partially repair the structure and supermicrostructure of the tubal epithelium. The concentration of tumor necrosis factor (TNF)-α in the untreated control group was significantly higher than that in the wild-type group (*P* = 0.015). The concentration of TNF-α in the local treatment group was significantly lower than that in the untreated control group (*P* = 0.011). The expression of oviductal glycoprotein (OVGP) and OVGP mRNA in the wild-type group was significantly higher than those in the untreated control group (*P* = 0.024 and *P* = 0.013, respectively). The litter size of the treatment group was 2 ± 2.39 kits, which was higher than that of the untreated control group (*P* = 0.035).

**Conclusion:**

Chronic inflammation can destroy the structure of the oviduct and the supermicrostructure of epithelial cells as well as leading to infertility. WJMSC transplantation therapy in rabbits with chronic salpingitis partially restored fertility. WJMSCs also repaired the structure of the tubal epithelium subjected to chronic inflammation, decreased the level of inflammatory factors, and partially restored the secretion level of OVGP.

## Background

Tubal infertility has long been considered the major cause of female infertility. Salpingitis and/or pelvic inflammation is one of the most important factors in tubal infertility. Severe salpingitis can damage the fallopian tube (FT) mucosa, and pelvic inflammation damages the structure of the oviduct, which might result in fimbria adhesion, distal tube obstruction, and hydrosalpinx [1]. Thus it is necessary to determine new methods to repair the reproduction function of the oviduct, thereby allowing gametes to combine and grow in vivo under natural circumstances.

Mesenchymal stem cells (MSCs) [2] are used in cell therapy and regenerative medicine because they are easily isolated and acquired, exhibit rapid expansion in culture, can be used in autologous transplantation, and exhibit significant paracrine effects [3]. Wharton’s jelly-derived MSCs (WJMSCs) possess distinct advantages, such as accessibility, painless donation procedures, and high separation rates [4]. Moreover, WJMSCs do not express major histocompatibility complex (MHC) II [5] and exhibit low immunogenicity and little to no MHC I expression [6]. WJMSCs exhibit a higher proliferation capacity and lower expression of CD106, HLA-ABC, and HLA-DR than MSCs from bone [7, 8]. In a previous study, we injected a WJMSC suspension into rats with chronic salpingitis using different methods [9]. The results demonstrated that WJMSCs could decrease the serum level of inflammatory factors and restore the structure and function of the oviduct and recover fertility.

Therefore, our study established a chronic salpingitis model in New Zealand rabbits and transplanted WJMSCs using different methods to treat this infection. The therapeutic effect was evaluated by assessing morphology, inflammatory factors, proteinology, and pregnancy outcomes.

## Methods

### Isolation, culture, and identification of WJMSCs

Human umbilical cord tissue was obtained from healthy and full-term infants who were born via social-factor cesarean section. HbsAg, anti-HIV, CMV-IgM, syphilis, mycoplasma, and chlamydia tests were negative. The umbilical cord tissue was washed with D-Hanks balanced salt solution (BSS). The umbilical veins, umbilical artery, and the outer membrane of the umbilical tissue were dislodged. Wharton’s jelly was removed and cut into 1 × 1 × 1 mm tissue blocks. These tissue blocks were resuspended in 0.075% type I collagenase and incubated at 37 °C for 10–14 h with magnetic stirrers. The digested mixture was washed and diluted in D-Hanks BSS, and the suspensions were centrifuged at 1500 rpm for 5 min at room temperature to obtain a cell pellet. The pellet was washed and centrifuged three times in D-Hanks BSS. The pellet was washed and resuspended in a growth medium containing Dulbecco’s modified Eagle’s medium (DMEM) and 10% fetal bovine serum (FBS), and cultured in a 37 °C incubator with 5% CO_2_. The growth medium was renewed every 3 days. Cells that attached to the dish were fusiform fibroblasts and were 80% confluent in approximately 1 week. Flow cytometry was used to detect the presence of CD73, CD90, and CD105 and the absence of CD34 and CD45 to determine which cells were WJMSCs. The cell viability in our study was 95–98%. The cells were passaged to the fourth passage and diluted with a sterile saline solution to 1 × 10^6^/ml on the day of study. All materials were manufactured and provided by the Cord Blood Bank of Guangdong Province, China, on the day of use.

### Model bacterial strain

A lyophilized (ATCC25922) strain was inoculated in a fresh beef infusion broth and cultured in an incubator (37 °C) for 24 h. The mixture was centrifuged at 1000 rpm for 10 min at room temperature to obtain precipitates. The precipitates were washed three times in phosphate-buffered saline (PBS) and diluted with a sterile saline solution to a 3 × 10^8^/ml *Escherichia coli* suspension.

### Establishment of the animal model in the pre-experiments

Ten female New Zealand rabbits (4–5 months old, nonpregnant, weighing 2500 ± 250 g) were provided by the Animal Experiment Center of the Guangzhou University of Chinese Medicine (qualification no. CV20130015). All rabbits were fed in the animal experiment center for approximately 1 week to adapt to their environment.

**Fig. 1:**
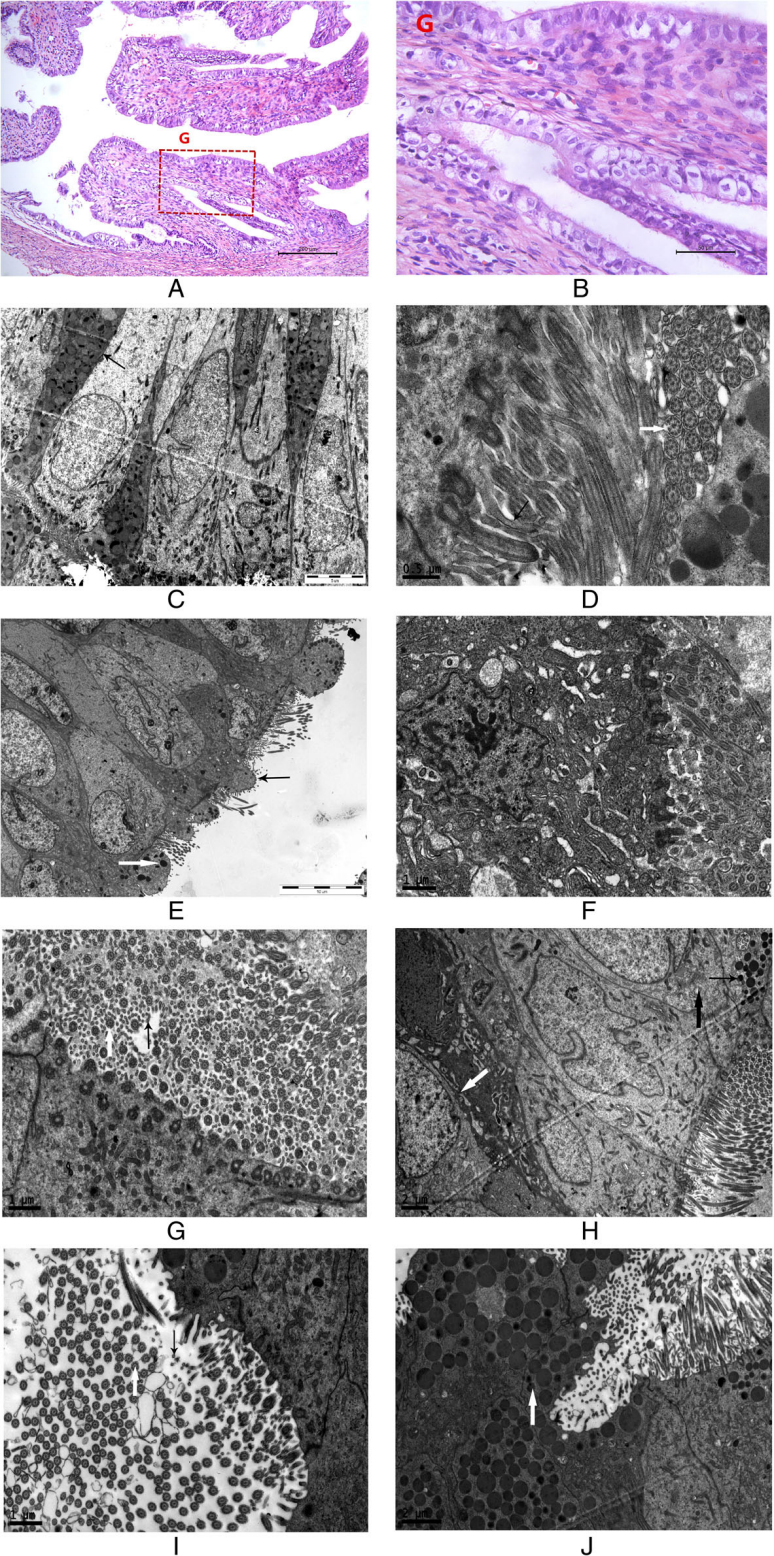
Hematoxylin and eosing staining (**a**: 100×; **b**: 400× enlargement of the area G in **a**) of the ampulla of a fallopian tube 15 days after *Escherichia coli* suspension intubation. We observed interstitial proliferation and lymphocyte infiltration (*n* = 2). **c** Secretory epithelium and ciliated cells showed an opposite arrangement. Many secretion granules and mitochondria were observed in the secretory cells (*black arrow*) at 3700× in the wild-type group (*n* = 6). **d** Cilia (*white arrow*) and microvilli (*black arrow*) were abundant, and the cilia showed the typical 9 × 2 + 2 arrangement of microtubules at 23,000× in the wild-type group (*n* = 6). **e** Microvilli and cilia were sparse (*black arrow*), and secretory granules decreased significantly (*white arrow*) at 2550× in the untreated control group (*n* = 10). **f** Incomplete cytomembranes, disappearing microvilli, and deciduous cilium were observed in some epithelial cells. Some organelles, such as swollen endoplasmic reticula and mitochondria, projected into the oviduct lumen at 9700× in the untreated control group (*n* = 10). **g** Most microvilli (*black arrow*) and cilia (*white arrow*) renewed. The typical 9 × 2 + 2 arrangement of the microtubules was clear at 9700× in the vein + local treatment group (*n* = 6). **h** Secretory granules remained reduced (*thin black arrow*). Swollen mitochondria (*thick black arrow*) and endoplasmic reticula (*white arrow*) were still observed in the epithelial cytoplasm at 3900× in the local vein + local treatment group (*n* = 6). **i** Microvilli (*black arrow*) and cilia (*white arrow*) were renewed. The typical 9 × 2 + 2 arrangement of microtubules was clear at 9700× in the local treatment group (*n* = 6). **j** Secretory granules (*white arrow*) were observed in secretory cells at 5800× in the local treatment group (*n* = 6)

Female rabbits were anesthetized using sodium pentobarbital and fixed to expose the vulva. Disposable sterile newborn sputum suction tubes were inserted 8–10 cm into the urogenital tract. The absence of fluid drawn demonstrated that the sputum suction tubes were inserted into the uterine cavity rather than the bladder. An *Escherichia coli* suspension (1 ml/kg, 3 × 10^8^/ml) was injected into the uterine cavity via sputum suction tubes, and the hips of the rabbit were elevated for 3 min. Two rabbits were humanely sacrificed 5, 10, 15, and 20 days after intubation. Two rabbits without *Escherichia coli* suspension injections were humanely sacrificed 20 days after human chorionic gonadotropin (HCG) injection to observe the morphological structure and obstruction rate of the FT. We sampled parts of the interstitial and ampulla tissues of the FTs of all rabbits to observe pathological changes using a light microscope. Pathological changes were assessed by analysis of three slides per animal sample, five random HPF per slide, and two rabbits per different sacrificing time. The pre-experiment demonstrated that the transvaginal intrauterine administration of a 3 × 10^8^/ml *Escherichia coli* suspension was sufficient to establish a chronic salpingitis model in female New Zealand rabbits. Salpingitis became a chronic inflammatory condition in 15 days (Fig. 1a and b).

### Experimental animals and grouping

Sixty pathogen-free female New Zealand rabbits (4–5 months old, nonpregnant, weighing 2500 ± 250 g) were provided by the Animal Experiment Center of the Guangzhou University of Chinese Medicine (qualification no. CV20130015). All experimental animals were divided into three groups: a wild-type group (*n* = 12), an untreated control group (*n* = 24), and a treatment group (*n* = 24). All female rabbits were injected with 80 IU HCG to synchronize their estrous cycles. Female rabbits in the untreated control and treatment groups were anesthetized using pentobarbital sodium. Disposable sterile newborn sputum suction tubes were inserted into the urogenital tract to instill an *Escherichia coli* suspension into the uterine cavity to establish the chronic salpingitis model.

Untreated control rabbits (*n* = 24) were perfused with an *Escherichia coli* suspension and randomly divided into the vein + local and local groups. Fifteen days after intubation, rabbits in the vein + local untreated control group were injected with 0.5 ml normal saline via the ear vein. Disposable sterile newborn sputum suction tubes were inserted into the urogenital tract of rabbits to perfuse 0.5 ml normal saline. This procedure was executed once per week for 3 weeks. Rabbits in the local untreated control group received 1 ml normal saline via the urogenital tract. This procedure was executed once per week for 3 weeks. Six rabbits in each group were randomly sacrificed 1 week after the last normal saline perfusion by an intravenous injection of a high dose of the anesthetic solution, and the oviducts and blood from abdominal aorta were sampled for examination. Six remaining rabbits in each group were injected with 80 IU HCG to stimulate ovulation and then paired with male rabbits for 30 days to observe fertility.

Rabbits in the treatment group (*n* = 24) were randomly assigned to the vein + local and local groups. Fifteen days after HCG injection, rabbits in the vein + local treatment group (*n* = 12) were injected with 0.5 ml of a 1 × 10^6^/ml WJMSC suspension via the ear vein. Disposable sterile newborn sputum suction tubes were inserted into the urogenital tract of rabbits to instill 0.5 ml of a 1 × 10^6^/ml WJMSC suspension. This procedure was executed once per week for 3 weeks. Rabbits in the local treatment group (*n* = 12) received 1.0 ml of a 1 × 10^6^/ml WJMSC suspension via the urogenital tract. This procedure was executed once per week for 3 weeks. Six rabbits in each group were humanely sacrificed 1 week after the last WJMSC perfusion, while the oviducts and blood from the abdominal aorta were sampled for examination. Six remaining rabbits in each group were injected with 80 IU HCG to stimulate ovulation and then paired with male rabbits for 30 days to observed fertility.

The wild-type group (*n* = 12) did not receive any treatment after HCG injection. Six rabbits were randomly selected and humanely sacrificed on experimental day 36, and the oviducts and blood from the abdominal aorta were sampled for examination. Six remaining rabbits in each group were injected with 80 IU HCG to stimulate ovulation and then paired with male rabbits for 30 days to observe fertility (Table 1).

**Table 1:** Treatments and experimental design

Group	Treatment	Samples	Fertility observation
Wild-type group (*n* = 12)	Receive no treatment after HCG injection	The oviducts and blood from the abdominal aorta of six rabbits in each group were sampled on experimental day 36	Six remaining rabbits in each group were injected with 80 IU HCG and paired with male rabbits for 30 days
Vein + local untreated control group (*n* = 12)	Ear vein and urogenital tract were instilled by normal saline, respectively, on experimental day 15, once per week for 3 weeks		
Local untreated control group (*n* = 12)	Urogenital tract was instilled by normal saline on experimental day 15, once per week for 3 weeks		
Vein + local treatment group (*n* = 12)	Ear vein and urogenital tract were instilled by 0.5 ml of a 1 × 10^6^/ml WJMSC suspension, respectively, on experimental day 15, once per week for 3 weeks		
Local treatment group (*n* = 12)	Urogenital tract was instilled by 1.0 ml of a 1 × 10^6^/ml WJMSC suspension on experimental day 15, once per week for 3 weeks		

*HCG* human chorionic gonadotropin, *WJMSC* Wharton’s jelly-derived mesenchymal stem cell

### Preparation of fallopian tubes for transmission electron microscopy

The FTs were sampled and cut into 1 × 1 × 1 mm blocks, immersed in a mixture of 2.5% glutaraldehyde stationary liquid in PBS (4 °C, pH 7.4, 0.1 M) for 24 h, followed by fixation in phosphate-buffered 1% osmium tetroxide for 2 h at room temperature. Afterwards, the FT samples were dehydrated in ascending concentrations of ethyl alcohol, infiltrated with a propylene oxide-Araldite mixture, and embedded in Araldite. Ultrathin sections were then stained with uranyl acetate and lead citrate. The ultrastructure of the oviduct was examined and photographed using a Tecnai G2 Spirit transmission electron microscope (TEM). Twenty-five electron micrographs per animal in each group were taken, and the images were processed using Adobe Photoshop 7.0 software.

### Expression detection of oviductal glycoprotein mRNA via real-time fluorescence quantitative RT-PCR

Total RNA was extracted using a Trizol reagent (Invitrogen, Carlsbad, CA, USA) according to the manufacturer’s instructions. The RNA samples were diluted and then examined via a microspectrophotometer, and an OD260/280 absorption ratio between 1.8 and 2.0 was used for further analysis. Total RNA samples were reverse-transcribed using Reverse Transcriptase M-MLV (RNase H-; TAKARA, JPN). Quantitative polymerase chain reaction (qPCR) was performed in a 20-μl volume containing SYBR® Premix Ex Taq™ (Tli RNaseH Plus; Takara, Japan), gene-specific primer, 2.0 μl cDNA, and 7.5 μl dH_2_O. The reactions were conducted with an initial denaturation step of 95 °C for 5 min, followed by 45 cycles of 95 °C for 3 s and 60 °C for 34 s. Each PCR reaction included a non-template negative control with nuclease-free water instead of cDNA. The relative expression of the target genes was analyzed using the 2^–ΔΔCT^ method: ΔCT = CT (a target gene) – CT (a reference gene). ΔΔCT = ΔCT (a target sample) – ΔCT (a reference sample). The results are presented as the fold-change of target gene expression in a target sample relative to a reference sample, normalized to 18S. The reference sample is the oviduct sample from one rabbit in the wild-type group in our study. The PCR product was sequenced to verify via BLAST. Each experiment was performed in three biological replicates. Specific primers for qPCR were designed by Sangon Biotech (Shanghai) Co. Ltd. The following primer sequences were used in the present study: oviductal glycoprotein (OVGP), forward primer (5’-GGATGTCTGAAGCACCCAGAGGT-3’), reverse primer (5’-AGGTCATCGTCATCTTGCCAGGG-3’); 18S forward primer (5’-GAATTCCCAGTAAGTGCGGGTCATA-3’), reverse primer (5’-CGAGGGCCTCACTAAACCATC-3’).

### Expression detection of oviductal glycoprotein via Western blot

The samples were homogenized in ice-cold RIPA buffer. The protein concentration was quantified using a BCA protein assay (Thermo Fisher Scientific, Rockford, USA). Equal amounts of protein (30 μg/lane) were subjected to 10% SDS-PAGE and subsequently transferred to polyvinylidene difluoride (PVDF; Millipore, Bedford, MA, USA) membranes. After blocking in 5% fat-free dry milk, the membranes with OVGP and GAPDH (inner reference) were incubated overnight at 4 °C with an anti-OVGP antibody (Santa Cruz Inc., USA) diluted 1:1000 and an anti-GAPDH antibody (Santa Cruz Inc.) separately. After washing, the membrane was incubated with horseradish peroxidase-linked donkey anti-goat IgG (1:3000, Beyotime Biotechnology, CHN) for 2 h. Bound antibodies were detected using an ECL detection system (Vazyme Biotech, China). The immunoreactive bands were quantified using Quantity One software (Bio-Rad Laboratories).

### Quantification of TNF-alpha by ELISA

Concentrations of tumor necrosis factor (TNF)-α were measured using an enzyme-linked immunosorbent assay (ELISA) according to the manufacturer's specifications (CUSABIO and CusAb, Wuhan, China). The sensitivity limit of the test was 19.5 pg/ml. All samples were measured in duplicate.

### Statistical methods

All data are presented as means ± standard errors. Data in this article were analyzed using one-way analysis of variance (ANOVA) and the least-significant difference (LSD) test (homogeneity of variance) or Tamhane’s T2 test (heterogeneity of variance), and a value of *P* ˂ 0.05 was considered significant. All data were analyzed using SPSS 13.0 statistical software (SPSS, Inc., Chicago, IL, USA). All bar charts were generated using GraphPad Prism 5.01 (GraphPad Software, Inc., La Jolla, CA, USA).

## Results

Two rabbits in the vein + local untreated control group died on days 20 and 27 of the experiment because of diarrhea. The other rabbits in the experiment lived. We did not obtain blood from the abdominal aorta of one rabbit in the vein + local untreated control group when it was humanely sacrificed. White, purulent secretions were observed from the vagina of two rabbits in the vein + local untreated control group on day 45 of the experiment. Five days later, the purulent secretions ceased without any intervening measure.

### Electron microscopy results

In the wild-type group, the secretory epithelium and ciliated cells had an opposite arrangement in the ampullary segment of the oviduct. Many secretion granules and mitochondria were observed in the secretory cells, and microvilli appeared on the cell surface. The ciliated cells were columnar, and the free surface of the cells was covered with cilia that were thicker and longer than microvilli. Every cilium showed the typical 9 × 2 + 2 arrangement of microtubules (i.e., a ring of 9 doublets plus 2 single microtubules in the center) and could repeat slapping movements. In the cytoplasm, sporadic rough endoplasmic reticula, ribosomes, and a few secretory granules were observed. The nuclei of epithelial cells were oval and showed low electron density.

We were able to clearly observe intercellular tight junctions.

Epithelial basement membranes were complete. Mesenchymal cells and smooth muscle cells in the submucosa showed an orderly arrangement. The nuclei of these cells were elongated. Abundant mitochondria were observed in the cells, and some of them were vacuoles. Some fiber was observed in the intercellular areas (Fig. 1c and d).

In the untreated control groups, the cilia of most epithelial cells in the oviduct decreased. The typical 9 × 2 + 2 arrangement of the microtubules became blurred. The microvilli of the secretory cells were sparse, and secretory granules decreased significantly. Cells appeared to be aging, had deep-dyed cytoplasm, were swelling, and had vacuolated mitochondria and autolysosomes. The electric density of the nuclei was high. Nucleus shrinkage and deformation, chromatin edge accumulation, and perinuclear space broadening were observed in some cells. Incomplete cytomembranes, disappearing microvilli, and deciduous cilia were observed in some epithelial cells. Certain organelles, such as swollen endoplasmic reticula and mitochondria, were projected into the oviduct lumen.

We were able to observe the intercellular tight junctions faintly.

Epithelial basement membranes were complete. Proliferous collagenous fiber around the mesenchymal cells was observed under the epithelia. Mesenchymal cells showed an irregular shape. Autolysosomes, lipid droplets, lipofuscin, and swelling mitochondria were observed in the cytoplasm. The electric density of most nuclei was high. Chromatin edge accumulation was observed in some cells (Fig. 1e and f).

In the vein + local treatment group, the cilia of most epithelial cells in the oviduct increased significantly. The typical 9 × 2 + 2 arrangement of the microtubules was clear. Most microvilli of the secretory cells renewed, but secretory granules remained reduced. Swollen mitochondria and endoplasmic reticula remained in the epithelial cytoplasm. Nucleus shrinkage and deformation, chromatin edge accumulation, and perinuclear space broadening was not observed in the cells.

We were able to clearly observe the intercellular tight junctions.

Epithelial basement membranes were complete. The proliferous collagenous fiber around the mesenchymal cells under the epithelium was less than that in the untreated control group. Mesenchymal cells showed an elongated and regular arrangement. Swelling mitochondria were still observed in the cytoplasm. The electric density of most nuclei was high. Chromatin edge accumulation was observed in some cells (Fig. 1g and h).

In the local treatment group, the cilia and microvilli of the epithelial cells were renewed, similar to those in the vein + local treatment group. Secretory granules were observed in secretory cells. In the epithelial cytoplasm, swollen mitochondria were reduced compared with those in the untreated control group. Many mitochondria and endoplasmic reticula were observed in the epithelial cytoplasm. Nucleus shrinkage and deformation, chromatin edge accumulation, and perinuclear space broadening were not observed in the cells.

We were able to clearly observe the intercellular tight junctions.

Epithelial basement membranes were complete. The proliferous collagenous fiber around the mesenchymal cells under the epithelium was reduced compared with that in the untreated control group. Capillary proliferation was observed in the mesenchymal layer. Mesenchymal cells had a regular arrangement. Swelling mitochondria were still observed in the cytoplasm. Chromatin edge accumulation was less than that in the untreated control group (Fig. 1i and j).

### Concentration of TNF-α of each group via ELISA

**Fig. 2:**
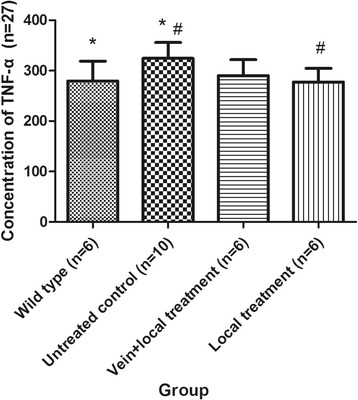
Concentration of tumor necrosis factor (*TNF*)-α in each group. *The concentration of TNF-α in the untreated control group was significantly higher than that in the wild-type group (*P* = 0.015). ^#^The concentration of TNF-α in the local treatment group was significantly higher than that in the untreated control group (*P* = 0.011). Data are presented as the mean ± SD

The concentration of TNF-α in the vein + local untreated control group was 321.78 ± 42.57 pg/ml, whereas in the local untreated control group it was 326.05 ± 28.70 pg/ml. No significant difference was found between these groups (*P* = 0.857). Given that the missing data from the vein + local untreated control group might have influenced the analysis and that no significant differences were found between the vein + local untreated control group and the local untreated control group, we combined these two untreated control groups. One-way ANOVA and LSD tests (homogeneity of variance, *P* = 0.891) were performed to examine the concentration of TNF-α in different groups. The concentrations of TNF-α in the different groups were significantly different (*P* = 0.030). The concentration of TNF-α in the untreated control group was significantly higher than that in the wild-type group (*P* = 0.015). The concentration of TNF-α in the local treatment group was significantly lower than that in the untreated control group (*P* = 0.011). The concentration of TNF-α in the vein + local treatment group was lower than that in the untreated control group; however, no significant differences were observed (*P* = 0.055). The concentration of TNF-α in the local treatment group was lower than that in the vein + local treatment group, but this difference was not significant (*P* = 0.512) (Table 2, Fig. 2).

**Table 2:** Concentration of TNF-α in each group

Group	*n*	TNF-α (pg/ml)	Compared with wild-type group	Compared with untreated control group	Compared with vein + local treatment group
			*P*1	*P*2	*P*3
Wild-type group	6	279.38 ± 39.52			
Untreated control group	9	324.63 ± 31.18	**0.015**		
Vein + local treatment group	6	290.06 ± 31.84	0.575	0.055	
Local treatment group	6	277.54 ± 26.91	0.923	**0.011**	0.512

Significant differences are indicated in bold typeface

*TNF* tumor necrosis factor

### Expression of OVGP in the rabbit oviduct of each group

#### Expression of OVGP in the rabbit oviduct of each group via Western blot

**Fig. 3:**
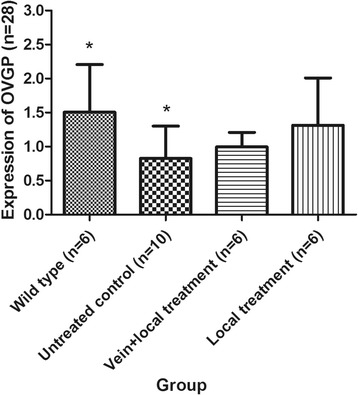
The expression of oviductal glycoprotein (*OVGP*) in each group. *The expression of OVGP in the wild-type group was approximately two times that of the untreated control group, and this difference was significant (*P* = 0.024). Data are presented as the mean ± SD

The expression of OVGP in the vein + local untreated control group was 0.90 ± 0.73, whereas that in the local untreated control group was 0.78 ± 0.28. No significant difference was found between these groups (*P* = 0.743). Given that the missing data from the vein + local untreated control group might have influenced the analysis and that no significant differences were found between the vein + local untreated control group and the local untreated control group, we combined these two untreated control groups. One-way ANOVA and LSD tests (homogeneity of variance, *P* = 0.080) were performed to examine the expression of OVGP in different groups. In our study, the expression of OVGP in the wild-type group was approximately twice that of the untreated control group, and this difference was significant (*P* = 0.024). The expression of OVGP in the vein + local treatment group and the local treatment group was higher than that in the untreated control group; however, no significant differences were observed (*P* = 0.554 and *P* = 0.097, respectively). The expression of OVGP in the local treatment group was higher than that in the vein + local treatment group, but this difference was not significant (*P* = 0.323) (Table 3, Fig. 3).

**Table 3:** Expression of OVGP in each group

Group	*n*	OVGP content	Compared with wild-type group	Compared with untreated control group	Compared with vein + local treatment group
			P1	P2	P3
Wild-type group	6	1.51 ± 0.70			
Untreated control group	10	0.83 ± 0.48	**0.024**		
Vein + local treatment group	6	0.99 ± 0.21	0.117	0.554	
Local treatment group	6	1.31 ± 0.69	0.543	0.097	0.323

Significant differences are indicated in bold typeface

*OVGP* oviductal glycoprotein

#### Expression of OVGP mRNA in the rabbit oviduct of each group via RT-PCR

**Fig. 4:**
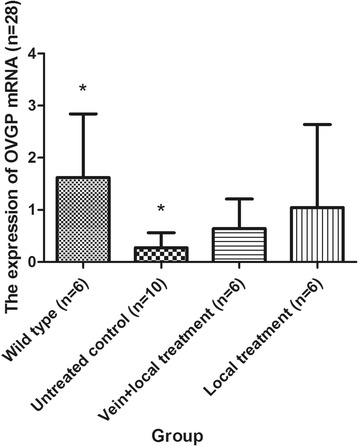
The expression of oviductal glycoprotein (*OVGP*) mRNA in each group. *The expression of OVGP mRNA in the wild-type group was approximately six times that in the untreated control group, and this difference was significant (*P* = 0.013). Data are presented as the mean ± SD

The expression of OVGP mRNA in the vein + local untreated control group was 0.27 ± 0.38, whereas the expression in the local untreated control group was 0.27 ± 0.26. No significant difference was found between these groups (*P* = 0.999). Given that the missing data from the vein + local untreated control group might influence the analysis and that no significant differences were found between the vein + local untreated control group and the local untreated control group, we combined these two untreated control groups. One-way ANOVA and LSD tests (homogeneity of variance, *P* = 0.063) were performed to examine the expression of OVGP mRNA in the different groups. In our study, the expression of OVGP mRNA in the wild-type group was approximately six times that in the untreated control group, and this difference was significant (*P* = 0.013). The expression of OVGP mRNA in the vein + local treatment group and the vein + local treatment group was higher than that in the untreated control group, but these differences were not significant (*P* = 0.465 and *P* = 0.135, respectively). The expression of OVGP mRNA in the local treatment group was approximately 100% higher than that in the vein + local treatment group, but this difference was not significant (*P* = 0.480) (Table 4, Fig. 4).

**Table 4:** The expression of OVGP mRNA in each group

Group	Number (n)	OVGP mRNA	Compared with wild-type group	Compared with untreated control group	Compared with vein + local treatment group
			P1	P2	P3
Wild-type group	6	1.62 ± 1.22			
Untreated control group	10	0.27 ± 0.29	**0.013**		
Vein + local treatment group	6	0.64 ± 0.57	0.094	0.465	
Local treatment group	6	1.05 ± 1.59	0.314	0.135	0.480

Significant differences are indicated in bold typeface

*OVGP* oviductal glycoprotein

### Litter size of New Zealand rabbits in each group

One-way ANOVA and Tamhane’s T2 test (heterogeneity of variance, *P* = 0.000) were performed to examine the litter size of the different groups. In the wild-type group, 5 of 6 rabbits became pregnant. The pregnancy rate was 83%, and the litter size was 6 ± 2.93 kits. No pregnancies were observed in the two untreated control groups. In the vein + local treatment group, 3 of 6 rabbits became pregnant. The pregnancy rate was 50%, and the litter size was 2 ± 1.76 kits. The litter size was more than that in the vein + local untreated control group, but this difference was not significant (*P* = 0.616). In the local treatment group, 3 of 6 rabbits became pregnant. The pregnancy rate was 50%, and the litter size was 3 ± 2.94 kits. The litter size was higher than that of the local untreated control group and the vein + local treatment group, but these differences were not significant (*P* = 0.552 and *P* = 0.996, respectively) (Table 5).

**Table 5:** Litter size of the New Zealand rabbits in each group

	1	2	3	4	5	6
Wild-type group	7	6	7	8	0	7
Vein + local untreated control group	0	0	0	0	0	0
Local untreated control group	0	0	0	0	0	0
Vein + local treatment group	2	4	0	0	0	3
Local treatment group	6	5	5	0	0	0

**Fig. 5:**
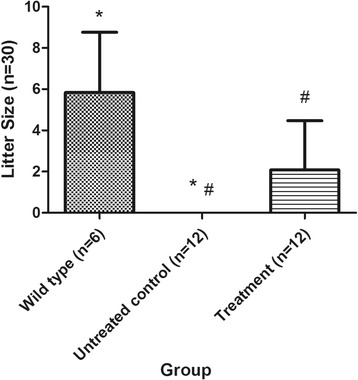
Litter size of the New Zealand rabbits in each group. *The litter size of the untreated control group was 0 ± 0.00 kits, which was less than that of the wild-type group (*P* = 0.014). ^#^The litter size of the treatment group was 2 ± 2.39 kits, which was more than that of the untreated control group (*P* = 0.035). Data are presented as the mean ± SD

Given that only 6 rabbits were assigned to each group (which might have influenced the statistical results) and that no significant differences were found between the untreated control groups or between the treatment groups, we combined the two untreated control groups into a single untreated control group, and we combined the two treatment groups into a single treatment group. One-way ANOVA and Tamhane’s T2 test (heterogeneity of variance, *P* = 0.000) were performed to examine the litter size of the different groups. We found that the litter size of the treatment group was 2 ± 2.39 kits, which was more than that of the untreated control group (*P* = 0.035). The litter size of the treatment group was still smaller than that of the wild-type group, but these differences were not significant (*P* = 0.073) (Table 6, Fig. 5).

**Table 6:** Pregnancy rate and litter size in each group

Group	Pregnancy rate	Litter size (*n*)	Compared with wild-type group	Compared with untreated control group
			P1	P2
Wild-type group	83%	6 ± 2.93		
Untreated control group	0%	0 ± 0.00	**0.014**	
Treatment group	50%	2 ± 2.39	0.073	**0.035**

Significant differences are indicated in bold typeface

## Discussion

### The influence of chronic inflammation on oviducts

Oviducts in mammals assume the important reproductive functions of egg transportation, sperm transportation, sperm activation, and early embryonic development. A normal ciliary beat, the secretion of epithelial cells, and oviduct wall peristalsis all play important roles in reproductive function [10, 11]. Importantly, the normal structure of microtubules in the cilia directly influence ciliary beat. For example, the oviduct epidermis of patients with Kartagener’s syndrome was biopsied, and the number of cilia in every epithelial cell was less than 20% of the typical amount, and central microtubules were missing. This abnormal structure resulted in primary infertility because of ciliary motility disorders [12]. Microvilli are also an important structure of the epithelial cells in the oviduct. They can increase the surface area of cells and the reception of stimuli on the cells as well as adhering to sperm to avoid polyspermy [13]. Epithelial cells can also secrete; oviduct fluid consists of specific oviductal glycoproteins, hormones, and growth factors, and can offer developmental support to gametes and early embryos as well as help with the self-defense of the oviduct [14]. TNF-α is a multifunctional pro-inflammatory cytokine that is associated with various pathological processes, such as proliferation, apoptosis, immunoregulation, and inflammation. Additionally, TNF-α is closely associated with immunity of female genital organs [15, 16]. When genital organs are infected or under inflammatory stimulation, high levels of TNF-α will aggravate the mucous epithelium of genital organs [17, 18].

In our study, incomplete epithelial cell membranes, missing microvilli, cilia desquamating, and organelle dysfunction, such as swollen endoplasmic reticula, the mitochondria projecting into the oviduct lumen, and proliferating mesenchymal cells, were observed via an electron microscope. TNF-α concentrations in the untreated control group were higher than that in the wild-type group. In addition, twelve rabbits in the untreated control groups were not pregnant. These results demonstrated that, with chronic inflammation, epithelial cells aged and the functions of the cilia and secretions were damaged, resulting in changes to the microenvironment of egg-sperm binding and the transportation frustration of the sperm and egg. The change in structure and function of the oviduct finally leads to tubal infertility.

### The therapeutic effect of WJMSCs on chronic tubal inflammatory infertility

New Zealand rabbits are often used in animal experiments because they have strong reproductive capacity and survivability. In our study, 5 of the 6 rabbits in the wild-type group became pregnant in 30 days. The pregnancy rate was 83%, and litter size was 6 ± 2.93. The litter size in our study matched that of a previous study, but the pregnancy rates were not in agreement with previous reports of 100% [19]. This difference might be because of different raising environments and seasons.

After three vaginal perfusion treatments using 10^6^ WJMSCs, three of six rabbits became pregnant in the local treatment group. The pregnancy rate was 50%, and the litter size was 3 ± 2.94 kits. After three intravenous plus vaginal perfusion treatments, three of six rabbits became pregnant in the vein + local treatment group. The pregnancy rate was 50%, and the litter size was 2 ± 1.76 kits. The pregnancy rates and litter sizes of the vein + local treatment group and the local treatment group were higher than those of the vein + local untreated control group and the local untreated control group, but no significant differences were observed. The lack of significant differences might have been caused by 1) the small sample size or 2) the early postcoital time. Previous studies have shown that no human cells were found in the body of a fetal lamb until 13 months after MSC transplantation [20]. Another study demonstrated that MSC transplantation via intrathecal injection improves the motor function of patients with cerebral palsy, and the curative effect was striking 6 months after transplantation [21]. Therefore, we conclude that, after transplantation in vivo, WJMSCs require a relatively long time to survive and produce an effect. In our study, rabbits in the treatment group were paired with male rabbits 1 week after the last WJMSC perfusion. The WJMSCs in our study might not have been able to display their full effect, resulting in no significant differences. In our next study, we will delay the postcoital time of the rabbits.

Given that only 6 rabbits were included in each group (which might have influenced the statistical results) and no significant differences were found between the untreated control groups or between the treatment groups, we combined the two untreated control groups into a single untreated control group and the two treatment groups into a single treatment group. Subsequently, we found that the litter size of the treatment group was 2 ± 2.39 kits, which was higher than that of the untreated control group (*P* = 0.035). This result demonstrates that WJMSCs show a therapeutic effect on chronic tubal inflammatory infertility and can restore fertility, at least partially. This therapeutic effect might be caused by epithelial cell growth promotion via secretion [22, 23] and possible differentiation into epithelial cells [24, 25]. Through these mechanisms, the basic structure and secretion function were restored. However, the litter size of the treatment group was smaller than that of the wild-type group, although these differences were not significant (*P* = 0.073). This result shows that the therapeutic effect of WJMSCs on chronic tubal inflammatory infertility is limited. Whether intrauterine combined with intraperitoneal administration can improve tubal and pelvic inflammation or whether other methods used to increase the local colonization of WJMSCs are more effective at treating tubal infertility requires additional research.

### The reparative effect of WJMSCs on tubal epithelia with chronic inflammation

Firstly, our study showed that the concentration of TNF-α in the local treatment group was significantly higher than that in the untreated control group (*P* = 0.011). The concentration of TNF-α in the vein + local treatment groups was higher than that in the untreated control group; however, no significant differences were observed (*P* = 0.055). These results demonstrated that WJMSCs could significantly decrease the level of pro-inflammatory factors to play an important anti-inflammatory role [26, 27].

The secretory function of tubal epithelial cells plays an important role in reproductive and developmental processes. In an in-vitro study, sperm and ovum co-cultured in tubal epithelial cells showed a greater fertility rate and blastula quantity and quality than under single-cultured conditions [28, 29]. The oviduct fluid secreted by tubal epithelial cells includes proteins, hormones, and growth factors. In addition, the proportion and ingredients vary with the level of estrogen and progesterone as well as different secretory sections [30]. Oviductal glycoprotein, which is synthesized and secreted by oviduct epithelial secretory cells, is the major protein in oviduct fluid. Many previous studies have shown that OVGP can combine with oocytes, sperm, and early embryos. These combinations can improve the capacitation, mobility, and fertilizing capacity of sperm [31–35]. Based on these important functions of OVGP in reproduction and secretion via tubal epithelial cells, the secretion volume of OVGP partially reflects the secretory function of tubal epithelial cells.

Our study detected the expression of OVGP using Western blot. The results showed that the expression of OVGP in the wild-type group was approximately twice that in the untreated control group, and this difference was significant (*P* = 0.024). The expression of OVGP in the vein + local treatment group and local treatment group after three periods was higher than that in the untreated control group; however, no significant differences were observed (*P* = 0.554 and *P* = 0.097, respectively), most likely because of the small sample size. However, these results likely demonstrate that WJMSCs can recover secretory function. The mechanism of the anti-inflammatory effect of WJMSCs can be divided into two parts. First, WJMSCs promote epithelial cell growth via secretion, such as the increased secretion of IL-10 and the inhibitory effect of pro-inflammatory factors as shown by our study, which improved the microenvironment of the injured sections [22, 23]. Second, WJMSCs may directly differentiate into epithelial cells. This mechanism was demonstrated by previous studies [24, 25].

To test whether the repair process of WJMSCs on the secretory function of tubal epithelial cells started with regulation at the transcriptional level, we used real-time fluorescence qRT-PCR to test the expression of OVGP mRNA. We found that the expression of OVGP mRNA in the wild-type group was approximately six times that in the untreated control group, and the difference was significant (*P* = 0.013). The expression of OVGP mRNA in the vein + local treatment group and the local treatment group were higher than those in the untreated control group, but these differences were not significant (*P* = 0.465 and *P* = 0.135, respectively). We considered that many factors influenced the expression of OVGP mRNA and that of OVGP (e.g., the variation in estrogen) [36]. Therefore, great individual variability exists, and larger sample sizes are needed. In addition, cell repair is a long process [37]. In future studies, we will use a relatively longer time after the last perfusion of WJMSCs.

## Conclusions

Chronic inflammation can destroy the structure of the oviduct and the supermicrostructure of epithelial cells as well as lead to infertility. WJMSC transplantation therapy in rabbits with chronic salpingitis helped partially restore fertility. WJMSCs also repaired the structure of the tubal epithelium subjected to chronic inflammation, decreased the level of inflammatory factors, and partially restored the secretion level of OVGP.

## Abbreviations

ANOVA: Analysis of variance
BSS: Balanced salt solution
ELISA: Enzyme-linked immunosorbent assay
FT: Fallopian tube
HCG: Human chorionic gonadotropin
LSD: Least-significant difference
MHC: Major histocompatibility complex
MSC: Mesenchymal stem cell
OVGP: Oviductal glycoprotein
PBS: Phosphate-buffered saline
qPCR: Quantitative polymerase chain reaction
TEM: Transmission electron microscope
TNF: Tumor necrosis factor
WJMSC: Wharton’s jelly-derived mesenchymal stem cell

## References

[1] Dun EC, Nezhat CH (2012). Tubal factor infertility: diagnosis and management in the era of assisted reproductive technology. Obstet Gynecol Clin North Am.

[2] Friedenstein AJ, Latzinik NW, Grosheva AG, Gorskaya UF (1982). Marrow microenvironment transfer by heterotopic transplantation of freshly isolated and cultured cells in porous sponges. Exp Hematol.

[3] Tan X, Gong YZ, Wu P, Liao DF, Zheng XL (2014). Mesenchymal stem cell-derived microparticles: a promising therapeutic strategy. Int J Mol Sci.

[4] Lu LL, Liu YJ, Yang SG, Zhao QJ, Wang X, Gong W (2006). Isolation and characterization of human umbilical cord mesenchymal stem cells with hematopoiesis-supportive function and other potentials. Haematologica.

[5] Gnoth C, Godehardt E, Frank-Herrmann P, Friol K, Tigges J, Freundl G (2005). Definition and prevalence of subfertility and infertility. Hum Reprod Hum Reprod.

[6] Cohen TS, Prince AS (2013). Activation of inflammasome signaling mediates pathology of acute P. aeruginosa pneumonia. J Clin Invest.

[7] Weiss ML, Medicetty S, Bledsoe AR, Rachakatla RS, Choi M, Merchav S (2006). Human umbilical cord matrix stem cells: preliminary characterization and effect of transplantation in a rodent model of Parkinson's disease. Stem Cells.

[8] Karahuseyinoglu S, Cinar O, Kilic E, Kara F, Akay GG, Demiralp DO (2007). Biology of stem cells in human umbilical cord stroma: in situ and in vitro surveys. Stem Cells.

[9] Luo HJ, Xiao XM, Zhou J, Wei W (2015). Therapeutic influence of intraperitoneal injection of Wharton's jelly-derived mesenchymal stem cells on oviduct function and fertility in rats with acute and chronic salpingitis. Genet Mol Res.

[10] Duran M, Ustunyurt E, Kosus A, Kosus N, Turhan N, Hizli D (2014). Does vitamin E prevent tubal damage caused by smoking? A light microscopy and animal study. Eur J Obstet Gynecol Reprod Biol.

[11] Lyons RA, Saridogan E, Djahanbakhch O (2006). The reproductive significance of human Fallopian tube cilia. Hum Reprod Update.

[12] Halbert SA, Patton DL, Zarutskie PW, Soules MR (1997). Function and structure of cilia in the fallopian tube of an infertile woman with Kartagener's syndrome. Hum Reprod.

[13] Hunter RH (2005). The Fallopian tubes in domestic mammals: how vital is their physiological activity?. Reprod Nutr Dev.

[14] Coy P, García-Vázquez FA, Visconti PE, Avilés M. Roles of the oviduct in mammalian fertilization. Reproduction. 2012;144(6):649-60. 10.1530/REP-12-0279 PMC4022750 23028122

[15] Morales P, Reyes P, Vargas M, Rios M, Imarai M, Cardenas H (2006). Infection of human fallopian tube epithelial cells with Neisseria gonorrhoeae protects cells from tumor necrosis factor alpha-induced apoptosis. Infect Immun.

[16] Oróstica ML1, Zuñiga LM, Utz D, Parada-Bustamante A, Velásquez LA, Cardenas H, et al. Tumour necrosis factor-α is the signal induced by mating to shutdown a 2-methoxyestradiol nongenomic action necessary to accelerate oviductal egg transport in the rat. Reproduction. 2013;145(2):109-17. 10.1530/REP-12-0389 23148087

[17] McGee ZA, Jensen RL, Clemens CM, Taylor-Robinson D, Johnson AP, Gregg CR (1999). Gonococcal infection of human fallopian tube mucosa in organ culture: relationship of mucosal tissue TNF-alpha concentration to sloughing of ciliated cells. Sex Transm Dis.

[18] Velasquez L, García K, Morales F, Heckels JE, Orihuela P, Rodas PI (2012). Neisseria gonorrhoeae pilus attenuates cytokine response of human fallopian tube explants. J Biomed Biotechnol.

[19] Xin G, Du J, Zhang J, Xu Y (2014). Novel reversible permanent contraception: an animal experiment of embedding contraceptive surgery in the fimbriated extremity of the fallopian. J Obstet Gynaecol Res.

[20] Saito T, Kuang JQ, Bittira B, Al-Khaldi A, Chiu RC (2002). Xenotransplant cardiac chimera: immune tolerance of adult stem cells. Ann Thorac Surg.

[21] Wang X, Cheng H, Hua R, Yang J, Dai G, Zhang Z (2013). Effects of bone marrow mesenchymal stromal cells on gross motor function measure scores of children with cerebral palsy: a preliminary clinical study. Cytotherapy.

[22] Gonzalez-Rey E, Anderson P, González MA, Rico L, Büscher D, Delgado M (2009). Human adult stem cells derived from adipose tissue protect against experimental colitis and sepsis. Gut.

[23] Wang H, Yang YF, Zhao L, Xiao FJ, Zhang QW, Wen ML (2013). Hepatocyte growth factor gene-modified mesenchymal stem cells reduce radiation-induced lung injury. Hum Gene Ther.

[24] Koh SH, Kim KS, Choi MR, Jung KH, Park KS, Chai YG (2008). Imp lantation of human umbilical cord-derived mesenchymal stem cells as a neuroprotective therapy for ischemic stroke in rats. Brain Res.

[25] Ding DC, Shyu WC, Chiang MF, Lin SZ, Chang YC, Wang HJ (2007). Enhancement of neuroplasticity through upregulation of beta1-integrin in human umbilical cord-derived stromal cell implanted stroke model. Neurobiol Dis.

[26] Ortiz LA, Dutreil M, Fattman C, Pandey AC, Torres G, Go K (2007). Interleukin 1 receptor antagonist mediates the antiinflammatory and antifibrotic effect of mesenchymal stem cells during lung injury. Proc Natl Acad Sci U S A.

[27] Chen W, Zhu F, Guo GH, Zhan JH (2011). Effect of bone marrow mesenchymal stem cells entransplantment on secretion of inflammatory cytokine in the early stages of smoke inhalation injury in rabbits. Zhongguo Wei Zhong Bing Ji Jiu Yi Xue.

[28] Aviles M, Gutierrez-Adan A, Coy P (2010). Oviductal secretions: will they be key factors for the future ARTs?. Mol Hum Reprod.

[29] Tan XW, Ma SF, Yu JN, Zhang X, Lan GC, Liu XY (2007). Effects of species and cellular activity of oviductal epithelial cells on their dialogue with co-cultured mouse embryos. Cell Tissue Res.

[30] Buhi WC (2002). Characterization and biological roles of oviduct-specific, oestrogen-dependent glycoprotein. Reproduction.

[31] Killian GJ (2004). Evidence for the role of oviduct secretions in sperm function, fertilization and embryo development. Anim Reprod Sci.

[32] Mccauley TC, Buhi WC, Wu GM, Mao J, Caamano JN, Didion BA (2003). Oviduct-specific glycoprotein modulates sperm-zona binding and improves efficiency of porcine fertilization in vitro. Biol Reprod.

[33] Merchan M, Peiro R, Santacreu MA, Francino O, Folch JM (2007). Rabbit oviductal glycoprotein 1 gene: genomic organization polymorphism analysis and mRNA expression. Mol Reprod Dev.

[34] Killian G (2011). Physiology and endocrinology symposium: evidence that oviduct secretions influence sperm function: a retrospective view for livestock. J Anim Sci.

[35] Pradeep MA, Jagadeesh J, De AK, Kaushik JK, Malakar D, Kumar S (2011). Purification, sequence characterization and effect of goat oviduct-specific glycoprotein on in vitro embryo development. Theriogenology.

[36] Briton-Jones C, Lok IH, Cheung CK, Chiu TT, Cheung LP, Haines C (2004). Estradiol regulation of oviductin/oviduct-specific glycoprotein messenger ribonucleic acid expression in human oviduct mucosal cells in vitro. Fertil Steril.

[37] Levicar N, Pai M, Habib NA, Tait P, Jiao LR, Marley SB (2008). Long-term clinical results of autologous infusion of mobilized adult bone marrow derived CD34+ cells in patients with chronic liver disease. Cell Prolif.

